# High-mobility group box 1 in acute kidney injury

**DOI:** 10.3389/fphar.2025.1618971

**Published:** 2025-07-14

**Authors:** Xuan Li, Guilin Jing, Zhentao Guo, Zhaoan Guo

**Affiliations:** ^1^ The First School of Clinical Medicine, Shandong University of Traditional Chinese Medicine, Jinan, Shandong, China; ^2^ Department of Nephrology, Affiliated Hospital of Shandong Second Medical University, Weifang, Shandong, China; ^3^ Department of Nephrology, Sunshine Union Hospital, Weifang, China; ^4^ Department of Nephrology, Shandong University of Traditional Chinese Medicine Affiliated Hospital, Jinan, Shandong, China

**Keywords:** acute kidney injury, HMGB1, inflammation, oxidative stress, ferroptosis, TLR4

## Abstract

Acute kidney injury (AKI) remains a major clinical concern owing to its association with elevated morbidity and mortality. The nuclear protein high-mobility group box protein 1 (HMGB1), recognized for its evolutionary conservation, has emerged as a key mediator in AKI pathogenesis. Upon cellular injury, HMGB1 translocate into the extracellular space, where it operates as a damage-associated molecular patterns molecule. Its release intensifies inflammatory responses, exacerbates oxidative stress, and triggers ferroptosis. Furthermore, HMGB1 engages receptors such as TLRs and RAGE, ultimately contributing to various forms of regulated cell death. This review comprehensively summarizes the biological characteristics, regulatory mechanisms, and pathological roles of HMGB1 in AKI. It highlights HMGB1’s central role in sepsis-associated AKI, ischemia-reperfusion injury, cisplatin-induced nephrotoxicity, and contrast-induced nephropathy. Moreover, HMGB1 demonstrates promising potential as a diagnostic and prognostic biomarker due to its early release and strong correlation with disease severity and outcomes. Targeting HMGB1 through natural compounds, small molecules, microRNAs, or specific antibodies shows therapeutic promise in preclinical models by attenuating inflammation, oxidative damage, and cell death. Future studies focusing on clinical validation and combination strategies may further establish HMGB1 as a diagnostic, prognostic, and therapeutic target, providing new avenues for improving AKI management and patient outcomes.

## 1 Introduction

Acute kidney injury (AKI) commonly manifests as a sudden impairment of kidney function, significantly elevating the likelihood of morbidity and mortality (Pérez-Aizpurua et al., 2024; Zarbock et al., 2024). Based on a global meta-analysis of hospital-based studies applying the Kidney Disease: Improving Global Outcomes (KDIGO) creatinine-based criteria for AKI, morbidity has been reported at 33.7% in children and 21.6% in adults (Ostermann et al., 2025). Mortality rates are similarly significant, amounting to 13.8% in pediatric cases and 23.9% among adults (Ostermann et al., 2025). Although supportive care has advanced, AKI continues to pose a significant global health burden due to its insidious onset, delayed diagnosis, and lack of effective therapeutic interventions (Li X. L. et al., 2023; Chen J. J. et al., 2024). Conventional diagnostic indicators, including serum creatinine and urine output, are often inadequate as they respond late to renal injury and fail to detect subclinical damage, limiting their utility in guiding early intervention (Allinson et al., 2023; Feng et al., 2023; Robinson et al., 2023). Despite the emergence of novel biomarkers, such as KIM-1 and NGAL, their diagnostic performance remains inadequate in many clinical settings, highlighting the pressing need to explore more effective alternatives (Lathiya et al., 2024; Ostermann et al., 2024; Rossiter et al., 2024; Zhao et al., 2025).

Numerous studies have shown that renal diseases including AKI and chronic kidney disease is related to many molecular mechanisms such as renin-angiotensin system, inflammation, pyroptosis, and TGF-β1/Smad signalling pathways (Fu and Dong, 2023; Miao et al., 2023; Lee et al., 2024; Wang H. et al., 2024) as well as microbial dysbiosis and metabolite disorder (Krukowski et al., 2023; Patschan et al., 2023; Miao et al., 2024a; Miao et al., 2024b; Yang K. et al., 2024). Serving as a critical regulator, high-mobility group box protein 1 (HMGB1) drives inflammation and cellular injury in multiple organ systems, with the kidney frequently affected (Liu T. et al., 2023; Datta et al., 2024b). In addition to its nuclear role in chromatin organization, HMGB1 acts as a DAMP molecule upon release from necrotic or stressed cells. It amplifies inflammatory pathways, promotes oxidative stress, and induces cell death by interacting with receptors such as TLRs and RAGE, ultimately driving tissue damage progression (Yang et al., 2020; Zhao Z. B. et al., 2023; Datta et al., 2024a). Its involvement has been documented in acute inflammatory conditions, including acute respiratory distress syndrome, myocardial infarction, and cerebral ischemia, highlighting its universal role in organ damage and repair (Young et al., 2023; Zheng et al., 2024).

In AKI, HMGB1 demonstrates unique advantages as both a biomarker and a therapeutic target. Studies reveal that HMGB1 is rapidly released into circulation following kidney injury, correlating closely with disease severity and outcomes (Matsuura et al., 2023). Unlike traditional markers, HMGB1 offers the potential for earlier detection of renal damage and provides mechanistic insights into its pathogenesis. Furthermore, preclinical evidence supports the feasibility of HMGB1-targeted therapies in mitigating inflammation, oxidative stress, and cell death, offering hope for improved AKI management (Chen et al., 2011a; Lau et al., 2014; Zhao Z. et al., 2023).

This article summarizes recent advances in understanding the role of HMGB1 in AKI, emphasizing its related molecular pathways. Specifically, we will explore the molecular mechanisms by which HMGB1 contributes to AKI pathogenesis, its potential as a diagnostic and prognostic biomarker, and emerging therapeutic strategies targeting HMGB1. By bridging preclinical evidence with clinical perspectives, this article seeks to highlight the translational potential of HMGB1 in improving AKI diagnosis, treatment, and prognosis, ultimately advancing the management of this life-threatening condition.

## 2 Biological functions of HMGB1

The HMGB family, belonging to the high-mobility group proteins, is the most abundant and widely studied member of this protein superfamily. To date, four mammalian members of the HMGB family have been characterized: HMGB1, HMGB2, HMGB3, and HMGB4, with HMGB1 being the most abundantly expressed. First identified in 1973, HMGB1 is a non-histone nuclear protein characterized by its rapid electrophoretic mobility (Goodwin et al., 1973; Chen et al., 2022). Its evolutionary significance is highlighted by the 99% amino acid sequence identity between humans and rodents (Andersson and Harris, 2010; Dong et al., 2022). HMGB1 is indispensable for survival, as systemic deletion of this protein in mice leads to fatal hypoglycemia shortly after birth (Calogero et al., 1999). Under cellular stress or damage, HMGB1 is swiftly released from the nucleus into the cytoplasm, with its intracellular levels rising markedly within seconds (Sapojnikova et al., 2005).

### 2.1 Structure and localization of HMGB1

**FIGURE 1:**
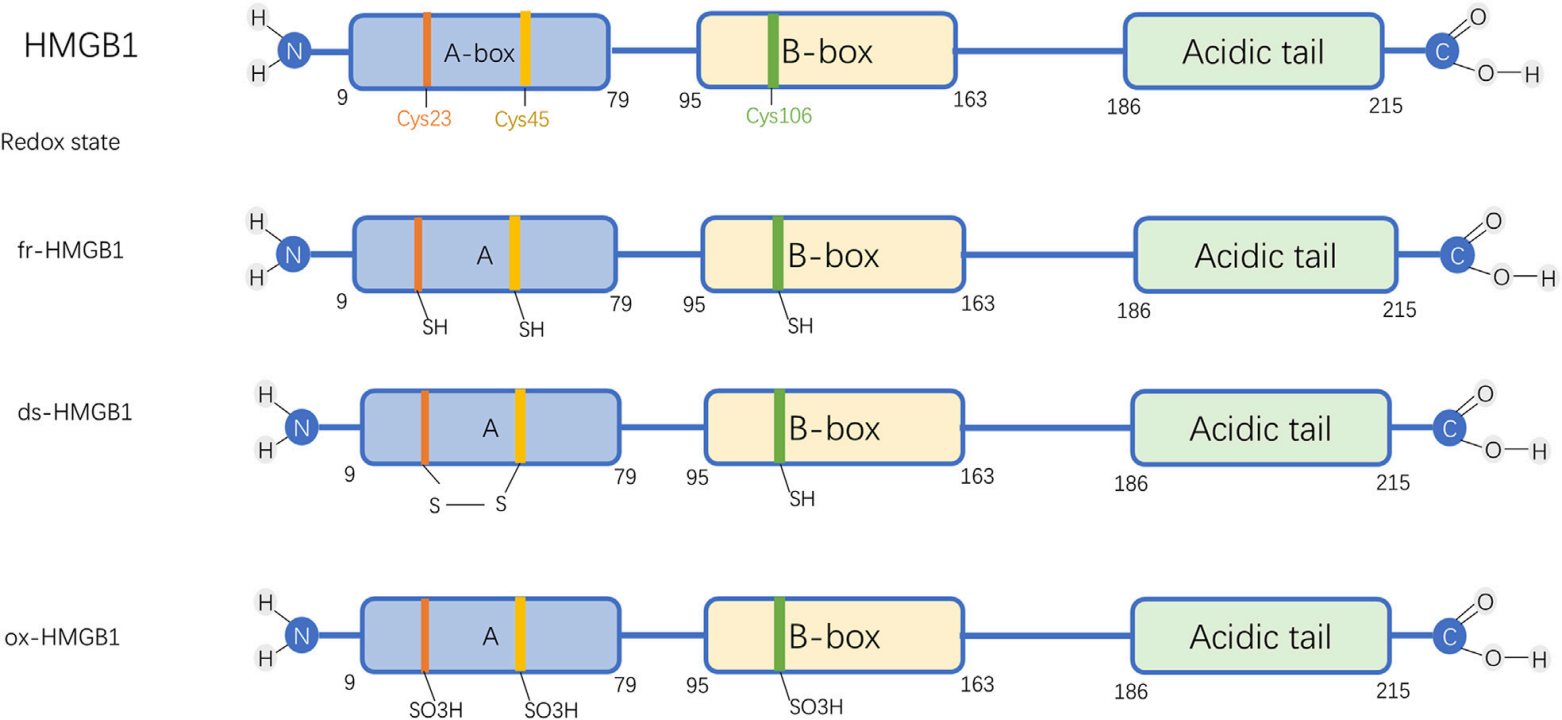
Molecular Structure and Functional Forms of HMGB1. Abbreviations: HMGB1, High-mobility group box protein 1; fr-HMGB1, Fully reduced HMGB1; ds-HMGB1, Disulfide HMGB1; ox-HMGB1, Fully oxidized HMGB1.

Composed of 215 amino acids, HMGB1 features two conserved DNA-binding regions known as the A-box and B-box, along with an acidic, negatively charged tail located at the C-terminus, and an essential N-terminal domain (Chen et al., 2022). Additionally, it possesses three redox-sensitive cysteine sites: C23, C45, and C106 (Figure 1). Three cysteine residues in HMGB1 (C23, C45, and C106) are sensitive to redox changes. Notably, C23 and C45 are capable of forming an intramolecular disulfide bond, whereas C106 predominantly exists in a reduced state. The redox state of these residues dictates HMGB1’s three isoforms: the fully reduced form (fr-HMGB1) with all thiol groups intact; the disulfide form (ds-HMGB1) featuring a C23-C45 bond; and the fully oxidized form (ox-HMGB1), also known as sulfonyl HMGB1.

The fully reduced form of HMGB1 interacts with chemokines to facilitate immune cell recruitment and contribute to tissue repair. The disulfide form stimulates immune cells, inducing cytokines and chemokines release and exhibiting enhanced affinity for CRM1-mediated nuclear export (Kwak et al., 2019). In contrast, fully oxidized HMGB1 loses both chemokine and cytokine activities (Chen et al., 2022). The reversible shift between fr-HMGB1 and ds-HMGB1 allows dynamic regulation, whereas further oxidative modification to ox-HMGB1 leads to an irreversible state (Avalos et al., 2010).

### 2.2 Functional significance and tissue distribution of HMGB1

HMGB1 exhibits abundant expression in renal tissues, with its involvement in AKI pathogenesis tightly governed by subcellular compartmentalization. In the nucleus, HMGB1 facilitates DNA repair and maintains nucleosome integrity and telomere stability (Martinotti et al., 2015; Voong et al., 2021; Liu T. et al., 2023). Retention of nuclear HMGB1 promotes peripheral B cell differentiation and augments both macrophage phagocytic activity and chemotactic responses (Avalos et al., 2010; Miao et al., 2020).

Cytoplasmic HMGB1 has been shown to regulate autophagic processes, alter mitochondrial dynamics, and impact apoptosis. Once released extracellularly, HMGB1 acts as a DAMP, driving immune cell differentiation, stimulating activation pathways, and enhancing cytokine production (Dumitriu et al., 2005). Furthermore, extracellular HMGB1 contributes to cell death by being internalized and transported to lysosomes, where it triggers downstream death pathways. This process leads to lysosomal membrane permeabilization (LMP), initiating downstream events that promote cell death (Deng et al., 2018; Feng et al., 2022).

### 2.3 HMGB1 modifications and regulation

HMGB1’s subcellular localization and functional activity are tightly regulated by multiple post-translational modifications (PTMs) (Stros, 2010). Acetylation strengthens the DNA-bending capability of HMGB1 while inhibiting its nuclear re-entry (Bonaldi et al., 2003; Pasheva et al., 2004). Pretreatment with resveratrol, a natural SIRT1 activator, decreases HMGB1 acetylation, thereby promoting its nuclear retention and attenuation of renal inflammation and tubular injury (Rabadi et al., 2015).

Methylation modifies HMGB1’s structure, impairs DNA binding, and facilitates passive cytoplasmic translocation for extracellular secretion (Ito et al., 2007). Phosphorylation alters HMGB1’s nuclear localization signals (NLS), thereby limiting its nuclear retention (Youn and Shin, 2006). Moreover, HMGB1 nuclear export to the extracellular milieu is enhanced by poly (ADP-ribose) polymerase (PARP) activity (Ditsworth et al., 2007). Poly-ADP-ribosylation of HMGB1 suppress gene transcription (Zhou et al., 2009; Guha et al., 2017), while disrupting macrophage efferocytosis, intensifying inflammation (Davis et al., 2012). Conversely, HMGB1 deficiency results in overactivation of PARP-1, exacerbating mitochondrial dysfunction and promoting cell death (Huang et al., 2014). Furthermore, PARP-1 activation facilitates HMGB1 release from proximal tubular cells (Kim, 2016).

Glycosylation is essential for HMGB1 secretion. Specifically, N-glycosylation decreases its DNA-binding affinity while promoting interaction with the nuclear export protein CRM1, facilitating cytoplasmic translocation and extracellular release (Kim et al., 2016). Moreover, N-glycosylation impairs HMGB1’s affinity for glycyrrhizin, a known HMGB1 inhibitor (Vergoten and Bailly, 2020). Emerging evidence indicates that O-GlcNAcylation modifies HMGB1, compromising its DNA repair capability (Balana et al., 2021). S-nitrosylation promotes HMGB1 secretion and amplifies its pro-inflammatory activity (Yang et al., 2022).

Ubiquitination drives HMGB1 degradation, leading to improved pathological outcomes (Sun et al., 2022). Ubiquitin-specific protease 12 (USP12) stabilizes HMGB1 by removing its ubiquitin chains, thereby promoting autophagy through direct interaction (Li et al., 2022). However, studies indicate that HMGB1 degradation is primarily mediated by the autophagy-lysosome pathway rather than ubiquitination. Autophagy activation and elevated CTSB levels facilitate HMGB1 degradation and promote its nuclear translocation (Zhou et al., 2020).

## 3 Mechanisms of HMGB1 in AKI

**FIGURE 2:**
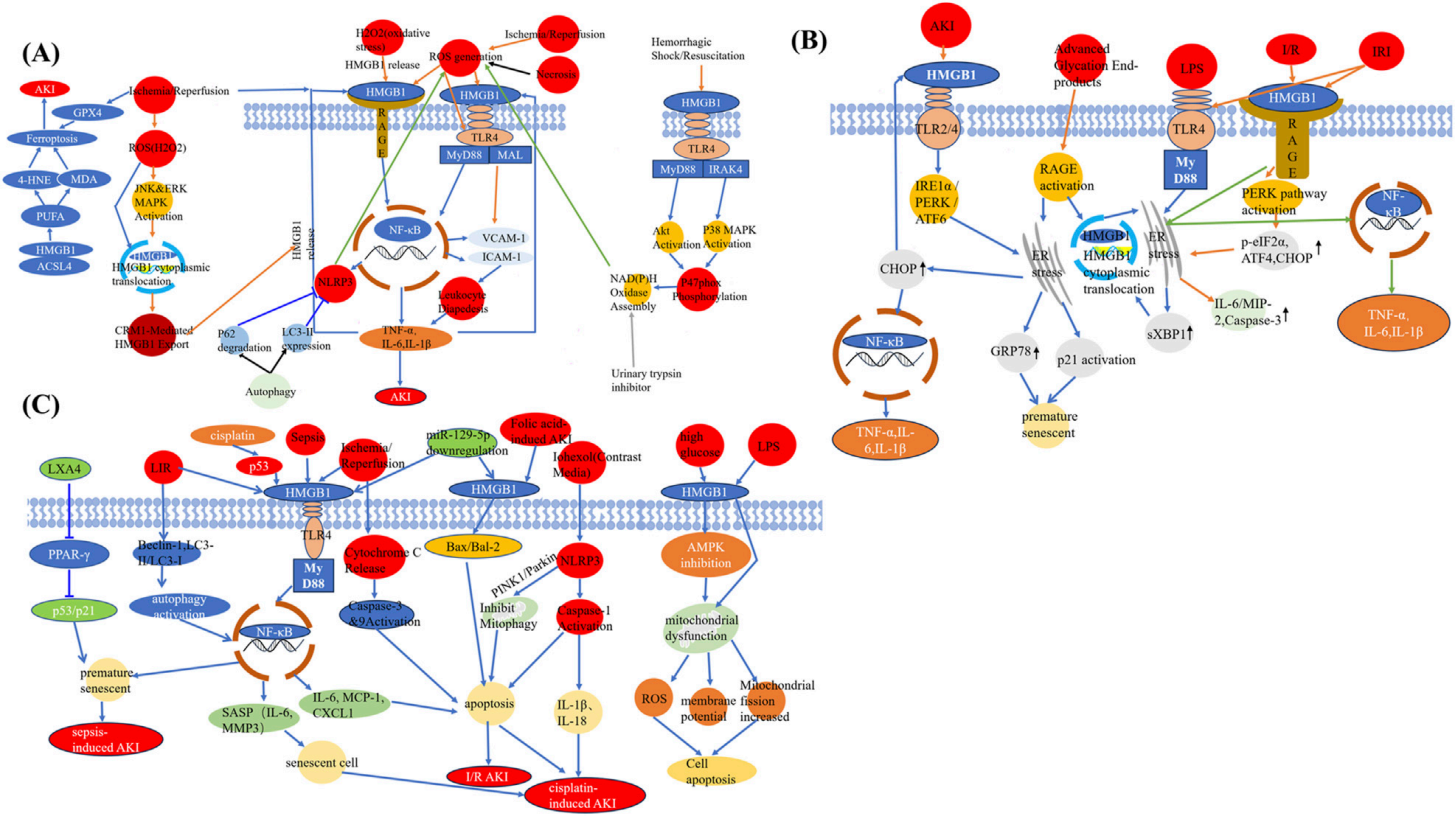
HMGB1 as a central mediator linking different injuries to AKI. **(A)** HMGB1 mediates ferroptosis and necroinflammation following ischemia-reperfusion, oxidative stress, and other AKI-inducing stimuli through interactions with ROS, MAPK, and autophagy pathways. **(B)** HMGB1 induces ER stress and inflammatory responses via TLR4, RAGE, and downstream effectors including PERK, CHOP, and NF-κB under various AKI-related insults. **(C)** HMGB1 promotes apoptosis, mitochondrial dysfunction, senescence, and pyroptosis in response to multiple pathological triggers such as sepsis, folic acid, contrast agents, and cisplatin. Abbreviations: AKI, Acute kidney injury; GPX4, Glutathione peroxidase 4; 4-HNE, 4-Hydroxynonenal; MDA, Malondialdehyde; PUFA, Polyunsaturated fatty acid; ACSL4, Acyl-CoA synthetase long-chain family member 4; ROS, Reactive oxygen species; JNK, c-Jun N-terminal kinase; ERK, Extracellular signal-regulated kinase; MAPK, Mitogen-activated protein kinase; HMGB1, High-mobility group box protein 1; CRM1, Chromosome region maintenance 1; RAGE, Receptor for advanced glycation end products; NLRP3, NOD-, LRR-, and pyrin domain-containing protein 3; NF-κB, Nuclear factor-kappa B; TNF-α, Tumor necrosis factor alpha; IL, Interleukin; TLR4, Toll-like receptor 4; MyD88, Myeloid differentiation primary response 88; MAL, MyD88-adapter-like protein; VCAM-1, Vascular cell adhesion molecule-1; ICAM-1, Intercellular adhesion molecule-1; IRAK4, Interleukin-1 receptor-associated kinase 4; LXA4, Lipoxin A4; PPAR-γ, Peroxisome proliferator-activated receptor-gamma; LIR, LC3-interacting region; SASP, Senescence-associated secretory phenotype; MMP3, Matrix metalloproteinase-3; MCP-1, Monocyte chemoattractant protein-1; CXCL1, C-X-C motif chemokine ligand 1; PINK1, PTEN-induced putative kinase 1; I/R, Ischemia/reperfusion; LPS, Lipopolysaccharide; AMPK, AMP-activated protein kinase; IRI, Ischemia-reperfusion injury; PERK, Protein kinase RNA-like ER kinase; IRE1α, Inositol-requiring enzyme 1 alpha; ATF, Activating transcription factor; CHOP, C/EBP homologous protein; sXBP1, Spliced X-box binding protein 1; MIP-2, Macrophage inflammatory protein-2; GRP78, Glucose-regulated protein 78; ER, Endoplasmic reticulum; p-eIF2α, Phosphorylated eukaryotic initiation factor 2 alpha.

HMGB1 is a central mediator in AKI, driving inflammation, oxidative stress, ferroptosis, and cell death. This section delineates the underlying molecular mechanisms, focusing on how HMGB1 interacts with specific receptors and signaling pathways to exacerbate kidney injury (Figure 2).

### 3.1 Amplifier of inflammation

HMGB1 acts as a DAMP by engaging pattern recognition receptors (PRRs) including TLRs and RAGE (Upadhyay and Batuman, 2022; Datta et al., 2024a). Upon activation, these receptors initiate downstream signaling cascades, particularly the MyD88-dependent NF-κB cascade.

HMGB1 binds to TLR4, activating MyD88-dependent NF-κB signaling. Both endothelial TLR4 and extracellular HMGB1 are essential for the expression of adhesion molecules CD54 and CD62E (Chen et al., 2011b). Adhesion molecules are upregulated on renal endothelial surfaces during the initial 4 h of reperfusion in mice with normal TLR4 expression, a response absent in TLR4-deficient mice (Chen et al., 2011b). As a result of this activation, elevated levels of pro-inflammatory cytokines—including TNF-α, IL-6, and IL-1β—are generated (de Oliveira Gomes et al., 2020; Zang et al., 2022). For instance, the release of HMGB1 during sepsis-induced AKI in rats is associated with a marked rise in pro-inflammatory factors emphasizing its contribution to inflammation (Hassan et al., 2024). Recent evidence by Datta et al. demonstrated that nicotine-induced podocyte injury is mediated by HMGB1-driven TLR4 activation. Pharmacological inhibition of HMGB1 (with glycyrrhizin) or TLR4 (with Resatorvid) effectively alleviated podocyte structural damage and inflammatory responses (Datta et al., 2024a). Chen et al. demonstrated HMGB1 from damaged renal cells triggers IL-6 production in leukocytes via TLR4 activation (Chen et al., 2011a).

RAGE-mediated signaling facilitates the recruitment of neutrophils and macrophages to damaged renal tissue, further exacerbating inflammation. Neutrophil infiltration into renal tissues amplifies inflammation and directly induces tubular injury by releasing reactive oxygen species (ROS) and proteases (Lau et al., 2014; Nakazawa et al., 2017).

Beyond cytokine production, HMGB1 drives endothelial activation by upregulating adhesion molecules like ICAM-1 and VCAM-1, thereby promoting leukocyte transmigration and intensifying the inflammatory response (Fiuza et al., 2003; Chen et al., 2011b; Park et al., 2018). This establishes a self-perpetuating cycle in which HMGB1-induced injury promotes further HMGB1 release, aggravating tissue damage.

### 3.2 Inducer of oxidative stress

Extensive research suggests that oxidative stress and inflammation play a critical role in kidney disease (Wang et al., 2023a; Kishi et al., 2024; Li et al., 2024; Miguel et al., 2025). HMGB1 exacerbates renal injury through mechanisms beyond inflammation. It directly elevates ROS production, disrupting mitochondrial function and inducing oxidative damage to tubular epithelial cells (Lau et al., 2014; Miyagawa et al., 2022). HMGB1 binding to TLR4 on endothelial and macrophages lead to excessive ROS production (Lu et al., 2012). Lu et al. reported that ischemia trigger ROS generation, leading to increased TLR4 expression on endothelial cells. Upon reperfusion for 4 h, damaged tubular cells release HMGB1, which subsequently binds to endothelial TLR4. This interaction promotes the upregulation of adhesion molecules on endothelial cells, facilitating leukocyte infiltration and aggravating maladaptive inflammation during ischemic AKI (Lu et al., 2012). In an *in vitro* ischemic AKI model, HMGB1 released from ROS-damaged proximal tubular cells, which subsequently activated TLR4-positive macrophages to secrete IL-6 (Lu et al., 2012). This finding indicates that HMGB1 derived from damaged cells stimulate leukocyte TLR4 to drive inflammation.

*In vivo*, a positive feedback loop involving HMGB1 contributes to ROS generation (Fan et al., 2007; Tang et al., 2007). *In vitro* studies have demonstrated that H2O2 stimulation promotes HMGB1 release from both macrophages and monocytes (Tang et al., 2007). Urinary trypsin inhibitor attenuated plasma HMGB1, which showed a significant correlation with superoxide anion radicals. This effect was likely mediated by suppressing superoxide anion radical generation, thereby interrupting the vicious cycle between ROS and HMGB1 during endotoxemia (Tanaka et al., 2010). HMGB1 has been implicated in enhancing the susceptibility of tubular cells to persistent oxidative stress (Zhao Z. B. et al., 2023). When tubular cells were subjected to 1 mM H_2_O_2_ for 24 h to model prolonged oxidative stress postinjury, HMGB1-deficient cells exhibited greater resilience within 10 h of incubation (Zhao Z. B. et al., 2023).

Furthermore, recent studies indicate that NOD-like receptor protein 3 (NLRP3) inflammasome activation is pivotal in promoting inflammation, apoptosis, and tissue injury in various nephropathies, including CI-AKI (Lin et al., 2021; Akhter et al., 2022; Awad et al., 2024). HMGB1 interacts with TLR4, facilitating NF-κB nuclear translocation. Acting as a critical upstream regulator, NF-κB enhances NLRP3 transcription, while the activated NLRP3 promotes reactive oxygen species (ROS) production, ultimately resulting in cellular damage (Awad et al., 2024). Inhibition of HMGB1 and its receptor TLR4 in the kidney exerts both anti-inflammatory and antioxidant actions, thereby enhancing renal function (Akhter et al., 2022; Awad et al., 2024). Reducing NF-κB activity in renal cells further downregulates NLRP3 and IL-1β levels, thereby mitigating inflammation (Akhter et al., 2022; Awad et al., 2024).

### 3.3 Ferroptosis, necroptosis and HMGB1

Acyl-CoA synthetase long-chain family member 4 (ACSL4), a member of acyl-CoA synthetase protein long-chain family, is related to lipid synthesis and lipid peroxidation dependent iron death (Mishra et al., 2024; Wang B. et al., 2024). Zhi et al. found HMGB1 translocation to the cytoplasm promotes ferroptosis by interacting with ACSL4, thereby promoting lipid peroxidation and non-apoptotic cell death (Zhao Z. et al., 2023). Ferroptosis markers, such as malondialdehyde (MDA) and lipid ROS, are significantly elevated in AKI models with high HMGB1 expression, further linking this pathway to renal damage (Tan et al., 2017; Mohamed et al., 2020). HMGB1 is a driver of tubular necrosis. HMGB1 promotes the expression of key necroptosis mediators, particularly receptor-interacting protein kinase 3 (RIP3) and mixed lineage kinase domain-like protein (MLKL) (Guo et al., 2018). These mediators orchestrate regulated necrosis, a process characterized by membrane disruption and leakage of intracellular contents that exacerbate inflammation and tissue injury (Oh et al., 2017; Akhter et al., 2022). Experimental evidence suggests that suppressing HMGB1 effectively reduces high glucose induced apoptosis in bone marrow stromal cells by activating the AMPK pathway and improving mitochondrial function (Liu B. et al., 2021). Cell death mediated by HMGB1 after renal ischemia reperfusion injury can be attenuated by glycyrrhizic acid treatment (Lau et al., 2014). In addition, HMGB1 also served as a marker of senescent cells (Davalos et al., 2013), which was decrasecd by paricalcitol in CI-AKI (Bae et al., 2020). Upregulation of HMGB-1 in the renal tissue leads to early and later apoptosis of glomerular cells and tubular epithelial cells, accumulation of extracellular matrix and accelerating renal fibrosis (Zhang Y. et al., 2020). Phloretin and empagliflozin alleviate inflammation and apoptosis in diabetes related ischemic acute kidney injury by modulating HMGB1-mediated TLR4/MyD88/NF-κB pathway (Amin et al., 2023). Following LIR, significant elevations were observed in HMGB1, IL-6 and TNF-a levels, along with upregulated Beclin 1 levels and an elevated LC3-II to LC3-I ratio. Modulation of autophagy influences HMGB1 expression and the production of inflammatory cytokines. Treatment with autophagy activator elevated HMGB1 levels and impaired renal function, indicating that excessive autophagy may contribute to the progression of LIR-induced AKI (Liu Z. et al., 2023). Lipoxin A4 improves renal function in sepsis by disrupting the interaction between HMGB1 and premature senescence (Chen et al., 2021). The overexpression of MEG3, an imprinted gene situated on chromosome 14q32.3 and induced by I/R, promotes tubular epithelial cell apoptosis through modulation of the miR-129-5p/HMGB1 axis (Mao et al., 2021). Melatonin enhances renal regeneration in folic acid-induced AKI by preventing HMGB1 nuclear export into the cytoplasm of tubular epithelial cells (Zhu et al., 2017).

This multifaceted involvement in oxidative stress and cell death underscores HMGB1 as a key driver of AKI progression. HMGB1 has been shown to trigger epithelial–mesenchymal transition in tubular epithelial cells during lipopolysaccharide-induced AKI (Gao et al., 2021). Exposure to HMGB1 significantly aggravated mitochondrial injury and induced apoptosis in HK-2 cells (Gao et al., 2021).

Although HMGB1 was traditionally considered to remain confined within the nucleus during apoptosis, studies have shown that its oxidized form is readily released from the apoptotic cells (Kazama et al., 2008), contributing to immune tolerance (Bell et al., 2006). Moreover, after phagocytosis of apoptotic debris, macrophages actively secrete HMGB1 (Qin et al., 2006), thereby overturning the traditional view of apoptosis as a “silent” death. Additionally, the TNF-α/HMGB1 inflammation axis is critically involved in mediating pyroptosis during AKI (Wang et al., 2020).

The endoplasmic reticulum (ER), a crucial intracellular structure essential for protein biosynthesis, also serves as a key regulator of inflammatory responses (Hu et al., 2023; Yang et al., 2023; Cheng et al., 2024; Yang M. et al., 2024). In various kidney diseases, HMGB1 and its receptors have been implicated in triggering ER stress in renal tubular cells, consequently exacerbating the progression of renal injury (Liu et al., 2014; Ferre et al., 2019; Lai et al., 2021). During the onset of AKI, TLR4 expression markedly increases in renal endothelial cells outside the bone marrow, while damaged renal interstitial cells release substantial amounts of HMGB1 (Zhang et al., 2023). HMGB1-TLR4 interaction enhances the expression of intercellular adhesion molecules, facilitating inflammatory cell infiltration into the renal parenchyma and triggering early inflammatory responses in AKI (Michel and Menze, 2019). Conversely, suppressing HMGB1 reduces inflammatory responses and alleviates ER stress in renal tubular epithelial cells (Zhang G. Z. et al., 2020). Additionally, VASPIN protects against renal ischemia-reperfusion injury by suppressing HMGB1 expression, thereby diminishing both inflammation and ER stress in these cells (Zhang G. Z. et al., 2020).

Activation of TLR2 and TLR4 influences key ER stress pathways by regulating sensors including IRE1-α (inositol-requiring enzyme 1α), PERK (protein kinase R-like ER kinase), and ATF6 (activating transcription factor 6) (Shelke et al., 2023a). This regulation exacerbates pro-inflammatory cytokine release, aggravating renal injury (Shelke et al., 2023a). Notably, downstream adaptor proteins of TLR2 and TLR4 activate ER stress signaling, leading to the activation of C/EBP homologous protein (CHOP) and sXBP1 (spliced X-box binding protein 1), which in turn promotes HMGB1 translocation to the nucleus. This process increases HMGB1 release, which subsequently interacts with TLR2/4 and further activates MyD88 signaling pathway (Shelke et al., 2023a).

Reducing ER stress can attenuate renal tubular cell apoptosis and secondary necrosis, thereby limiting HMGB1 release—both passive and active—and disrupting the HMGB1/TLR4/NF-κB signaling axis to mitigate inflammation (Zhang et al., 2015). Furthermore, blocking the TLR4-mediated MyD88/NF-κB signaling cascade suppresses inflammation independently of ER stress, given that MyD88 as an upstream adapter in this pathway (Zeng et al., 2012).

## 4 HMGB1 in specific types of AKI

### 4.1 Sepsis-associated AKI (S-AKI)

Sepsis is a leading cause of AKI, characterized by systemic inflammation and endothelial dysfunction (Li J. C. et al., 2023; Peng et al., 2024). HMGB1 plays a central role in S-AKI by amplifying inflammatory responses. HMGB1 exacerbates kidney injury by activating inflammatory pathways through binding to receptors such as TLR4 and RAGE (Wei et al., 2019; Ludes et al., 2021). This interaction triggers MyD88-dependent NF-κB signaling, leading to NLRP3 inflammasome formation and excessive secretion of pro-inflammatory cytokines (Hassan et al., 2024). These inflammatory mediators amplify systemic and local inflammation, induce apoptosis in tubular epithelial cell, and worsen oxidative stress, thereby aggravating renal damage. In chronic kidney disease (CKD), impaired HMGB1 clearance further amplifies its pathogenic role during sepsis, exacerbating S-AKI severity (Leelahavanichkul et al., 2011). Recent studies have demonstrated that circTLK1 enhances HMGB1 expression by acting as a sponge for miR-106a-5p, consequently accelerating sepsis-induced AKI pathogenesis. Silencing HMGB1 restores the protective effects of circTLK1 knockdown diminished by the miR-106a-5p inhibition, underscoring the pivotal role of the circTLK1–miR-106a-5p–HMGB1 regulatory axis in sepsis-associated AKI (Xu et al., 2021). HMGB1 acetylation is essential for its cytoplasmic translocation and extracellular release from renal cells, promoting the progression of sepsis-associated AKI (SA-AKI). Moreover, HMGB1 interaction with SIRT1 at deacetylated lysine positions K28, K29, and K30 effectively dampens subsequent inflammatory responses. Collectively, the SIRT1-HMGB1 axis is critically involved in SA-AKI pathogenesis (Wei et al., 2019).

Elevated serum and urinary HMGB1 levels have been identified as effective biomarkers for S-AKI, demonstrating high diagnostic accuracy (AUC: 0.891) when combined, outperforming single-sample detection methods (Zang et al., 2022). Furthermore, elevated HMGB1 levels are strongly correlated with poor prognosis, as evidenced by higher concentrations in non-survivors compared to survivors, underscoring its prognostic value (de Oliveira Gomes et al., 2020).

**FIGURE 3:**
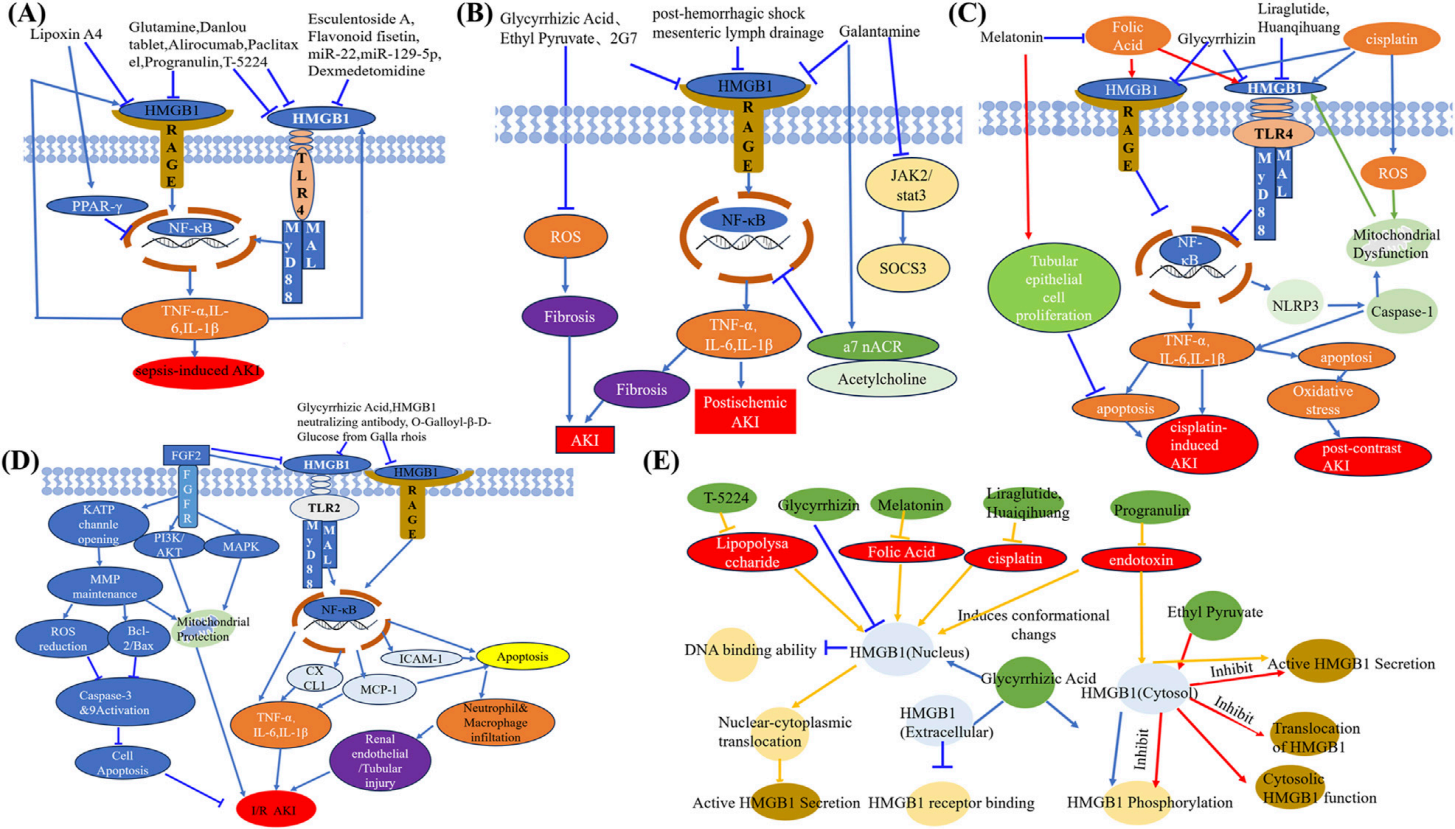
Therapeutic mechanisms of drugs acting on HMGB1 in AKI. **(A)** Inhibitors of HMGB1–RAGE and HMGB1–TLR4 pathways, including lipoxin A4, esculetin, paclitaxel, and others, ameliorate sepsis-induced AKI by blocking inflammatory cascades. **(B)** HMGB1 inhibitors such as glycyrrhizic acid, ethyl pyruvate, and galantamine alleviate post-ischemic AKI through suppression of ROS, fibrosis, and inflammatory signaling. **(C)** Drugs including melatonin, liraglutide, folic acid, and glycyrrhizin attenuate cisplatin- and contrast-induced AKI by regulating HMGB1-mediated mitochondrial injury, oxidative stress, and apoptosis. **(D)** Targeting HMGB1–TLR2/RAGE signaling through agents like FGF2, PI3K/AKT agonists, and natural compounds reduces inflammation, apoptosis, and I/R-induced AKI. **(E)** Schematic summary of compounds that inhibit HMGB1 translocation, phosphorylation, receptor binding, or cytosolic activity, thereby suppressing HMGB1-driven AKI pathology. Abbreviations: AKI, Acute kidney injury; HMGB1, High-mobility group box protein 1; RAGE, Receptor for advanced glycation end products; PPAR-γ, Peroxisome proliferator-activated receptor-gamma; TLR4, Toll-like receptor 4; MyD88, Myeloid differentiation primary response 88; MAL, MyD88-adapter-like protein; NF-κB, Nuclear factor-kappa B; TNF-α, Tumor necrosis factor alpha; IL, Interleukin; FGF2, Fibroblast growth factor 2; FGFR, Fibroblast growth factor receptor; KATP, ATP-sensitive potassium channel; ROS, Reactive oxygen species; MMP, Matrix metalloproteinase; PI3K, Phosphatidylinositol-3-kinase; MAPK, Mitogen-activated protein kinase; CXCL1, C-X-C motif chemokine ligand 1; MCP-1, Monocyte chemoattractant protein-1; ICAM-1, Intercellular adhesion molecule-1; JAK2, Janus kinase 2; STAT3, Signal transducer and activator of transcription 3; a7 nAChR, Alpha-7 nicotinic acetylcholine receptor; SOCS3, Suppressor of cytokine signaling 3; NLRP3, NOD-, LRR-, and pyrin domain-containing protein 3.

Therapeutic strategies targeting HMGB1 have shown promise in experimental models (Table 1; Figure 3). Increasing evidence has shown that natural products are extensively used to improve renal function and reduce kidney damage (Wang et al., 2023b; Guo et al., 2024; Vagopoulou et al., 2024; Zhong et al., 2024). Natural compounds such as Danlou Tablet and Isoliquiritigenin, along with small molecules like glutamine, reduce HMGB1 levels and attenuate its downstream inflammatory signaling, improving renal outcomes (Tang et al., 2018; Yu et al., 2024). Similarly, microRNAs like miR-22 and miR-370-3p downregulate HMGB1 expression and mitigate inflammation and apoptosis (Xu L. et al., 2020; Zhang et al., 2023) Biological agents, including anti-HMGB1 antibodies, Sivelestat, and T-5224, effectively suppress HMGB1-mediated inflammatory pathways, enhancing survival and renal function in preclinical studies (Hu et al., 2012; Ishida et al., 2015; Li et al., 2016). Dexmedetomidine attenuates AKI by activating α-2-adrenergic receptor, thereby suppressing pro-inflammatory cytokines in endotoxemic rats (Tan et al., 2015). Esculentoside A confers renal protection in septic rats by suppressing the HMGB1–TLR–NF-κB signaling cascade, thus alleviating inflammation (Sun G. et al., 2017). In a similar manner, glutamine ameliorates sepsis-induced kidney injury in mice through inhibition of HMGB1/TLR/NF-κB-driven inflammatory responses (Hu et al., 2012; Su et al., 2021). Additionally, miR-22 mitigates sepsis-associated AKI via downregulation of the HMGB1/TLR4/NF-κB pathway (Zhang et al., 2023), while miR-129-5p attenuates LPS-induced AKI by targeting the HMGB1/TLR/NF-κB axis (Huang et al., 2020). Mesenteric lymph drainage after hemorrhagic shock suppresses HMGB1 and RAGE expression in mouse renal (Liu et al., 2016). Galantamine alleviates inflammation by elevating acetylcholine (ACh) levels and regulating the NF-κB (p65)/HMGB1 axis (Ibrahim et al., 2018). Alirocumab reduces inflammatory responses in septic nephrotoxic rats through regulation of the PCSK9–HMGB1–NF-κB–NLRP3 signaling axis (Hassan et al., 2024). Flavonoid fisetin inhibit HMGB1 in S- AKI (Ren et al., 2020). Paclitaxel alleviates the sepsis-induced AKI through modulation of lnc-MALAT1–miR-370-3p–HMGB1 regulatory axis (Xu L. et al., 2020). PGRN deficiency aggravated renal injury and was associated with enhanced apoptosis, infiltration of inflammatory cells, elevated pro-inflammatory mediator production, and enhanced HMGB1 expression with cytoplasmic translocation in the kidney. Notably, rPGRN pretreatment prior to LPS exposure alleviated endotoxin-induced AKI in wild-type mice (Xu et al., 2016). T-5224, a selective c-Fos/activator protein-1 inhibitor, enhances survival by reducing serum HMGB1 levels in lethal lipopolysaccharide-induced AKI (Ishida et al., 2015). Using a rat model of early-stage sepsis, this research demonstrated that Xuebijing injection, when used as an adjuvant to antibiotic therapy, enhanced renal perfusion and oxygenation, reduced renal inflammation, and improved kidney function (Liu J. et al., 2021).

**TABLE 1:** Preclinical evidence of HMGB1-Targeted therapies in experimental AKI mode.

Drugs	Model	Cell	Pathway	AKI type	PMID
Danlou tablet	sepsis animal model	alleviated pro-inflammatory responses of LPS-stimulated macrophages	inhibit PARP1/HMGB1	S- AKI	38707378
Dexmedetomidine	endotoxemia rats		Through α-2-adrenergic receptor activation to decrease the inflammatory factors	LPS-induced AKI	26137237
Esculentoside A	sepsis animal model	kidney tubular epithelial cells	Inhibit HMGB1/TLR4/NF-kB pathway	S- AKI	28637971
FGF2	I/R rat model	-	inhibit HMBG1 and its downstream inflammatory cytokines	I/R AKI	28544332
Glutamine	sepsis mice		downregulate HMGB1/TLR/NF-kB pathway and RAGE	S- AKI	21921023
Glycyrrhizic acid	I/R mice	kidney tubular epithelial cells	HMGB1-mediated cell death and inflammation	I/R AKI	25059568
post-hemorrhagic shock mesenteric lymph drainage	A mouse hemorrhagic shock model	-	Inhibit HMGB1 and RAGE	AKI induced by severe hemorrhagic shock	26513053
1, 2, 3, 4, 6-Penta-O-Galloyl-β-D-Glucose from Galla rhois	I/R-AKI mice	renal tubular injury and microvascular inflammation	Decreased HMGB-1, MCP-1 and ICAM-1	I/R AKI	29754505
HMGB1 neutralizing antibody	I/R-AKI mice	kidney tubular epithelial cells	reduct tubular apoptosis and inflammation	I/R AKI	20679140 20847143
galantamine	Zymosan-Induced AKI in BALB/c Mice	-	Inhibit NF-κB (p65)/HMGB-1 signaling pathway	Zymosan-Induced AKI	30429582
Alirocumab	in rat model	-	PCSK9/HMGB1/NF-κB/NLRP3	S- AKI	38889644
Glycyrrhizic Acid	Mice	-	induce conformational changes interfering with HMGB1 DNA-binding ability in the nucleus, HMGB1 phosphorylation in the cytosol, and HMGB1 receptor binding ability in the extracellular space	postischemic AKI	36857499
Ethyl Pyruvate	Mice	-	inhibit translocation of HMGB1 from the nucleus, suppresses its functions in the cytosol and active HMGB1 secretion on cell activation	postischemic AKI	36857499
Flavonoid fisetin	sepsis mice	-	downregulate HMGB1/TLR/NF-kB pathway	S- AKI	31918290
lipoxin A4	sepsis mice		lock the crosstalk between inflammation and premature senescence in a PPAR-γ-dependent manner	S- AKI	33936050
Liraglutide	Mice and HK-2 cell		inhibit HMGB1 nuclear-cytoplasmic translocation and release	cisplatin-induced AKI	36746359
Melatonin	Male C57BL/6 mice	-	inhibition of nucleocytoplasmic translocation of HMGB1 in TECs	folic acid induced AKI	28469775
miR-22	AKI rats	-	HMGB1/TLR4/NF-κB pathway	S- AKI	35960478
miR-129-5p	AKI rats	-	HMGB1/TLR4/NF-κB pathway	LPS-induced AKI	33039954
Paclitaxel	AKI rats	-	lnc-MALAT1/miR-370-3p/HMGB1 axis	S- AKI	32998017
Huaiqihuang	mice	-	reduce cisplatin-induced release and nuclear-cytoplasmic translocation of HMGB1 and inactivated its downstream signaling molecules, TLR4 and NFκB, in renal tubular cells	cisplatin-induced nephrotoxicity	29743526
Progranulin	in WT mice		decreased apoptotic death, inflammatory cell infiltration, production of pro-inflammatory mediators and the expression and nucleus-to-cytoplasmic translocation of HMGB1	endotoxin-induced AKI	27367257
glycyrrhizin	PC-AKI rats		Inhibit HMGB1	post-contrast acute kidney injury	34341389
T-5224	rats		Inhibit HMGB1	lethal lipopolysaccharide-induced AKI	26579229

Abbreviations: AKI, acute kidney injury; S-AKI, Sepsis-Associated Acute Kidney Injury; I/R, Ischemia/Reperfusion; LPS, lipopolysaccharide; PARP1, Poly (ADP-ribose) polymerase 1; HMGB1, High-Mobility Group Box Protein 1; NF-κB, Nuclear Factor Kappa B; TLR4, Toll-Like Receptor 4; RAGE, receptor for advanced glycation end products; FGF2, Fibroblast Growth Factor 2; ICAM-1, Intercellular Adhesion Molecule 1; MCP-1, Monocyte Chemoattractant Protein 1; PCSK9, Proprotein Convertase Subtilisin/Kexin Type 9; NLRP3, NOD-, LRR-, and pyrin domain-containing protein 3; miR, MicroRNA; lncRNA, Long Non-Coding RNA; TEC, tubular epithelial cell; WT, wild type; PC-AKI, Post-Contrast Acute Kidney Injury.

### 4.2 Ischemia-reperfusion (I/R)-induced AKI

I/R injury is characterized by cellular damage induced during the recovery of blood circulation after an episode of ischemia or oxygen deprivation (Wu et al., 2023; Zhang et al., 2024).

I/R-induced AKI is a significant clinical challenge characterized by inflammation, oxidative stress, and cell death. Evidence from experimental studies indicates that HMGB1 levels rise significantly in both serum and renal tissues following ischemia-reperfusion injury (IRI) or nephrotoxin exposure, correlating strongly with kidney damage severity (Liu Z. et al., 2023; Zhao Z. et al., 2023). Lau et al. reported that HMGB1 protein expression progressively increased in kidneys between 4 and 24 h after ischemia, with immunohistochemistry detecting its presence in tubular cells as early as 4 h post-injury (Lau et al., 2014).

As same to S-AKI, HMGB1 also induced inflammation to amplify kidney injury through multiple pathways (Chen et al., 2011a; Lu et al., 2012). HMGB1 activates pattern recognition receptors such as TLRs and RAGE, triggering downstream pathways like NF-κB and MAPK signaling, and ultimately promoting the release of inflammatory mediators (Chen et al., 2011b; Lau et al., 2014; Miyagawa et al., 2022). This inflammatory cascade contributes to endothelial activation, leukocyte recruitment, and tubular injury, creating a vicious cycle that exacerbates tissue damage (Chen et al., 2011b; Lu et al., 2012; Gocze et al., 2018).

While HMGB1-mediated inflammation disrupts tissue repair, its modulation of autophagy and oxidative stress response also impacts the balance between injury and recovery (Lau et al., 2014; Tan et al., 2017; Tan et al., 2018). HMGB1 limits this regenerative capacity by increasing the cells’ susceptibility to oxidative stress. One study shows that HMGB1-deficient tubular cells exhibit greater resistance to prolonged oxidative stress, suggesting a negative impact of HMGB1 on kidney regeneration (Chen et al., 2011b).

Besides, ferroptosis is another critical mechanism in I/R-induced AKI (Chen Y. et al., 2024). HMGB1’s cytoplasmic translocation within tubular epithelial cells can trigger ferroptosis through interaction with ACSL4, further amplifying lipid peroxidation and cell death (Mohamed et al., 2020; Zhao Z. et al., 2023). For instance, studies have shown that limiting HMGB1’s nucleocytoplasmic translocation or neutralizing its extracellular activity can significantly reduce inflammation and ferroptosis, alleviating kidney injury (Lau et al., 2014; Tan et al., 2018; Wu et al., 2020).

Targeting HMGB1 presents a promising therapeutic avenue. Functional inhibitors like glycyrrhizic acid (GZA) and cyclic helix B peptide (CHBP) have demonstrated efficacy in preclinical models by reducing HMGB1 release and downstream signaling (Lau et al., 2014; Wu et al., 2020; Zhang Y. et al., 2020). Similarly, soluble RAGE (sRAGE) or neutralizing antibodies can disrupt HMGB1-TLR4/RAGE interactions, mitigating inflammatory cytokine production and tubular damage (Chen et al., 2011a; Lu et al., 2012; Miyagawa et al., 2022). Emerging therapies also focus on modulating HMGB1’s interaction with autophagy and ferroptosis pathways, offering additional strategies to limit its pathogenic effects (Tan et al., 2017; Zhao Z. et al., 2023). Fibroblast growth factor 2(FGF2) confers protection against I/RI and enhances survival in animal models by attenuating mitochondrial damage and suppressing HMGB1-driven inflammatory responses (Lau et al., 2014). Some publications uncovered that natural products improve kidney function by regulating autophagy and inflammation pathways (Shao et al., 2023; Ruan et al., 2024; Wang Y. N. et al., 2024; Ying et al., 2024). The compound 1,2,3,4,6-Penta-O-Galloyl-β-D-Glucose, extracted from Galla rhois, alleviates microvascular inflammation and tubular damage in AKI models through the downregulation of ICAM-1, MCP-1, and HMGB1 expression (Park et al., 2018). Antibodies against HMGB1 have been shown to ameliorate murine ischemic AKI (Wu et al., 2010; Li et al., 2011). The study reported that HMGB1 concentrations in peripheral blood were markedly elevated in patients with tubular cell injury (p = 0.019). Similarly, HMGB1 mRNA expression was markedly upregulated in the murine kidney (Gocze et al., 2018).

In summary, HMGB1 acts as a key driver of I/R-induced AKI by orchestrating inflammation, oxidative stress, and ferroptosis. Its central role in these processes highlights its potential as a therapeutic target, with strategies aimed at inhibiting its release, blocking receptor interactions, or modulating intracellular pathways showing promise in mitigating kidney injury and enhancing recovery.

### 4.3 Cisplatin-induced AKI

Cisplatin-induced AKI involves complex inflammatory and oxidative mechanisms (Li X. L. et al., 2023), where HMGB1 plays a pivotal role. Cisplatin exposure induces HMGB1 release from necrotic renal tubular epithelial cells, including HK-2 cells, into the extracellular space, thereby activating downstream inflammatory pathways involving TLR4/MyD88 and NF-κB signaling (Oh et al., 2017; Akhter et al., 2022). This aggravates tubular cell necrosis and apoptosis while promoting the production of pro-inflammatory cytokines, ultimately worsening kidney injury (Oh et al., 2017; Guo et al., 2018; Akhter et al., 2022).

Increasing evidence supports the therapeutic value of strategies targeting HMGB1. Rosmarinic acid (RA) and Huaiqihuang (HQH) extracts effectively inhibit HMGB1 release and downstream signaling, alleviating inflammation, oxidative stress, and tubular injury. These interventions also reduce markers of necrosis (e.g., RIP3 and MLKL) and improve renal function (Guo et al., 2018; Akhter et al., 2022). Additionally, herbal treatments such as Nelumbo nymphaea and Paeonia suffruticosa mitigate HMGB1-mediated damage by lowering HMGB1 levels and protecting proximal tubular cells from cisplatin-induced injury (Oh et al., 2017). Mesenchymal stem cells (MSCs) and mononuclear cells (MNCs) derived from human umbilical cord blood have also shown efficacy in reducing HMGB1 expression, decreasing Bax/Bcl-2 ratios, and alleviating apoptosis in renal tubular cells (Xu Q. et al., 2020). These treatments restore kidney function and attenuate inflammatory responses in cisplatin-induced AKI models. Liraglutide and its metabolites suppressed HMGB1 nuclear-cytoplasmic translocation and its subsequent release, thereby downregulating inflammatory cytokines and HMGB1 receptor expression. Exogenous recombinant HMGB1 (rHMGB1) markedly attenuated the protective effects of liraglutide and its metabolites against cisplatin-induced AKI (Xu et al., 2023).

The interplay between HMGB1 and other AKI biomarkers, such as NGAL and KIM-1, demonstrates a time- and dose-dependent relationship, where HMGB1 rises within hours post-cisplatin exposure, while NGAL and KIM-1 show delayed peaks (Oh et al., 2017). This interaction underscores the potential of combining HMGB1 with other biomarkers for early diagnosis and prognosis.

### 4.4 Contrast-induced AKI (CI-AKI)

Inflammation is critically involved in the onset and exacerbation of CI-AKI (Boccatonda et al., 2024; Deng et al., 2024). Notably, Guan et al. reported that exposure to contrast media significantly elevated HMGB1 release from renal tubular cells and triggered the upregulation of pro-inflammatory molecules, including TLR2, CXCR4, NF-κB, IL-6, and MCP-1 (Guan et al., 2017). This evidence highlights proximal tubular epithelial cells as key contributors to the inflammatory cascade during contrast-induced renal damage. By interacting with its receptors TLR2 and CXCR4, HMGB1 amplifies the inflammatory response, underscoring its role in the pathogenesis of CI-AKI (Guan et al., 2017).

Likewise, Hyewon et al. reported a significant rise in HMGB1 levels within in intracellular and serum compartments, accompanied by elevated oxidative stress markers, pro-inflammatory cytokines, and kidney injury biomarkers after contrast media exposure (Oh et al., 2021). Upon extracellular release, HMGB1 induces a cascade of multiple pro-inflammatory cytokines. These mediators tend to accumulate in renal tissues, thereby amplifying the inflammatory response (Andersson and Tracey, 2011; Yang et al., 2015).

As a programmed form of cell death, apoptosis is essential for preserving physiological homeostasis by removing unnecessary cells (Romano et al., 2008). The presence of apoptosis in renal cell injury caused by contrast agents highlights its importance in the development of CI-AKI (Romano et al., 2008; Luo et al., 2022). Increasing evidence suggests that TLR4 inhibition exerts protective effects against AKI. Hyperglycemia combined with ischemia/reperfusion injury has been shown to trigger apoptosis in renal tubular cells via the TLR4–MyD88–IK-β/α–NF-κB signaling pathway activation. This finding underscores the potential renoprotective effect of selectively inhibiting TLR4 (Shelke et al., 2023b). TLR4 activation is strongly linked to the induction of apoptotic cell death (Katare et al., 2020). Acting as a key downstream adaptor of TLR4, MyD88 initiates signaling cascades involving IκB/α and NF-κB, ultimately resulting in renal tubular cell apoptosis and inflammation (Shelke et al., 2023b).

Additionally, RAGE receptors are involved in apoptosis-related pathways (Huang et al., 2017). The HMGB1–RAGE pathway promotes apoptosis by inducing endoplasmic reticulum (ER) stress, primarily through activation of the PERK/eIF2α/ATF4 signaling cascade (He et al., 2022).

HMGB1 is recognized as a pivotal mediator in CI-AKI progression. Its interaction with TLR2, TLR4, and RAGE receptors activates NF-κB pathways, leading to increased secretion of pro-inflammatory cytokines (Zhou et al., 2021; Mo et al., 2024), thereby exacerbating renal tubular epithelial damage. This cascade exacerbates renal tubular epithelial damage and promotes apoptosis, contributing to the progression of CI-AKI. Furthermore, HMGB1 has shown a strong correlation with homocysteine (Hcy) levels, and their combined elevation serves as a predictive marker for CI-AKI risk and severity, underscoring its diagnostic value (Mo et al., 2022).

Therapeutic strategies targeting HMGB1 have shown promise in mitigating CI-AKI. Glycyrrhizin, a direct inhibitor of HMGB1, effectively reduces oxidative stress, inflammatory responses, and renal dysfunction by suppressing HMGB1 expression (Oh et al., 2021). Similarly, quercetin alleviates CI-AKI by suppressing the HIF-1α/lncRNA NEAT1/HMGB1 signaling axis, reducing both cell apoptosis and inflammation (Luo et al., 2022).

Owing to its pivotal involvement in CI-AKI progression and its regulatory effects on inflammation and apoptosis, HMGB1 has emerged as a promising therapeutic target. Agents such as glycyrrhizin, quercetin not only provide evidence for the efficacy of HMGB1-targeted therapies but also offer insights into the broader application of anti-inflammatory strategies in CI-AKI management (Oh et al., 2021; Luo et al., 2022). Therapeutic targeting of HMGB1 holds promise as a potential strategy to enhance clinical outcomes in CI-AKI patients (Mo et al., 2024).

Current evidence supports the efficacy of strategies targeting ER stress to reducing the risk of CI-AKI. According to Zhang et al., VASPIN suppresses HMGB1, activating the Nrf2/ARE/HO-1 signaling cascade, and subsequently inhibiting NF-κB activation. Consequently, this process mitigates both inflammatory responses and ER stress within renal tissues, leading to reduced kidney damage (Zhang G. Z. et al., 2020). Additionally, preventing stress-induced apoptosis has emerged as a promising approach for CI-AKI prevention, as confirmed by studies conducted on cellular and animal models (Peng et al., 2015; Sun Y. et al., 2017).

Atorvastatin mitigates CI-AKI by suppressing the TLR4–MyD88–NF-κB signaling pathway, enhancing renal tubular epithelial cell activity, reducing cellular damage, and inhibiting both pyroptosis and inflammatory responses (Yue et al., 2023). Cisplatin activated HMGB1-TLR4/MyD88 axis was also found to be downregulated with the rosmarinic acid treatment in CI-AKI (Akhter et al., 2022). Quercetin reduces cell damage and apoptosis in CI-AKI models by inhibiting HIF-1α-driven activation of the lncRNA NEAT1/HMGB1 axis (Luo et al., 2022).

## 5 HMGB1 as a biomarker for AKI

HMGB1 has demonstrated significant diagnostic and prognostic value across diverse AKI contexts. Compared to traditional biomarkers like serum creatinine, HMGB1 provides earlier detection of AKI (Table 2). Experimental studies reveal that HMGB1 levels rise within hours of kidney injury, significantly preceding creatinine elevation: A cross-sectional study revealed that serum HMGB1 concentrations were markedly higher in AKI patients compared to healthy individuals, CKD 5 patients and hemodialysis patients, with statistical significance (p < 0.001) (Zakiyanov et al., 2013).

**TABLE 2:** HMGB1 as a biomarker for AKI.

Patients	Methods	Diagnostic efficacy	PMID
patients with hepatitis B virus-related acute-on-chronic liver failure	ELISA	AUC = 0.72	36712653
cirrhotic patients	ELISA	discriminating nonsurvivors at 30 days: AUC 0.842	33061824
AKI patients treated with CRRT Plasma HMGB1 was measured on initiation	ELISA	associated with 90-day mortality (hazard ratio, 1.06; 95% CI, 1.04–1.08)	37336200
SAKI patient	ELISA	sensitivity 88%, specificity 87%, AUC 0.891	35985501
a smoke inhalation and burn swine model	ELISA	AKI: ROC 0.81, cut-off value 36.41 ng/mL	39534595
Variceal Bleeding in Patients with Advanced Chronic Liver Disease	ELISA	AKI: sensitivity 76.9%, specificity 72.2%, AUC 0.799	34618276
trauma patients	ELISA	sensitivity 48%, specificity 88%, AUC 0.69	38204152

Abbreviations: HMGB1, High-Mobility Group Box Protein 1; AKI, acute kidney injury; CRRT, continuous renal replacement therapy; SAKI, Sepsis-Associated Acute Kidney Injury; ELISA, Enzyme-Linked Immunosorbent Assay; AUC, area under the curve; ROC, receiver operating characteristic; CI, confidence interval; ng/mL, nanograms per milliliter.

Experimental studies further underscore its early diagnostic potential, as HMGB1 levels rise significantly 12 h before traditional markers like creatinine in burn and smoke inhalation models (Yang Z. et al., 2024). Measurement of HMGB1 levels 12 h after injury showed good predictive performance for AKI, yielding an AUC of 0.81 and identifying 36.41 ng/mL as the optimal threshold (Yang Z. et al., 2024). In trauma patients, HMGB1 levels at admission were predictive of AKI, with 59.7 μg/L identified as the threshold, demonstrating 48% sensitivity and 88% specificity, with an AUC of 0.69 (95% CI: 0.56–0.82) (Frelich et al., 2024).

Comparison with Other Biomarkers: In trauma-related AKI, although HMGB1 sensitivity is modest compared to NGAL, it remains a valuable addition to multimarker strategies (Frelich et al., 2024). Prognostic Value: In S-AKI, elevated serum and urine HMGB1 levels are strongly associated with AKI, with combined detection achieving high sensitivity (88%) and specificity (87%), enhancing diagnostic accuracy (Zang et al., 2022). Similarly, in cirrhotic patients, HMGB1 correlates with increased short-term mortality, showing an AUC of 0.842 for predicting 30-day outcomes (de Oliveira Gomes et al., 2020). Moreover, in critically ill patients requiring CRRT, HMGB1 levels (≥10 ng/mL) are linked to higher 90-day mortality and severe multi-organ dysfunction, highlighting its prognostic relevance (Matsuura et al., 2023). Yu et al. reported that HMGB1 was identified as a potential prognostic biomarker for AKI in HBV-induced acute-on-chronic liver failure (AUC = 0.727) (Liu et al., 2022). The optimal serum HMGB1 threshold for predicting AKI was 3,317.9 pg/mL, yielding 76.9% sensitivity and 72.2% specificity (AUC = 0.799) (Dos Santos Pinheiro et al., 2022).

Despite its promising diagnostic and prognostic potential, several limitations restrict the clinical application of HMGB1 in AKI. First, HMGB1 lacks disease specificity, as it is elevated in a wide range of inflammatory and ischemic conditions (Chen et al., 2022; Li et al., 2025). Second, the absence of standardized threshold values across studies-ranging from 36.4 ng/mL to over 3000 pg/mL-poses a challenge for clinical interpretation and cross-study comparison (Dos Santos Pinheiro et al., 2022; Frelich et al., 2024; Yang Z. et al., 2024). Additionally, differences in assay techniques and sample handling conditions introduce variability in measured concentrations. Notably, HMGB1 exists in multiple redox forms with distinct biological functions, yet most current studies overlook isoform specific effects (Shin et al., 2025).

Notably, significant scientific gaps remain in our understanding of HMGB1’s role in AKI. Few large-scale, multicenter studies have validated HMGB1’s clinical utility. The diagnostic performance of HMGB1 may be improved through multiplex approaches combining it with markers such as NGAL, TIMP-2, or KIM-1 (Yang Z. et al., 2024). Furthermore, although preclinical studies support therapeutic targeting of HMGB1-for instance, with glycyrrhizin or neutralizing antibodies-the clinical translation of such strategies remains limited (Yang et al., 2019; Paudel et al., 2020). Addressing these gaps is essential for establishing HMGB1 as a reliable biomarker and therapeutic target in AKI.

## 6 Conclusion

HMGB1 has emerged as a pivotal player in the pathogenesis, diagnosis, and prognosis of AKI. Acting as both a DAMP and a mediator of cellular injury, HMGB1 is involved in regulating inflammation, promoting oxidative stress, inducing ferroptosis, and triggering cell death.

Across diverse AKI contexts, including sepsis, ischemia-reperfusion injury, nephrotoxin exposure, and contrast, HMGB1 has demonstrated significant diagnostic and prognostic utility. Early elevations in HMGB1 provide a timely window for diagnosis, outperforming traditional markers like serum creatinine in sensitivity and specificity. Its correlation with disease severity and outcomes further highlights its prognostic relevance, particularly in critical scenarios such as continuous renal replacement therapy (CRRT)-dependent AKI.

Therapeutic strategies targeting HMGB1—ranging from natural compounds and small molecules to antibodies and receptor antagonists—have shown promising results in preclinical studies. These interventions not only mitigate inflammation and oxidative stress but also preserve renal function and enhance survival in experimental models. Despite these advances, challenges remain in translating these findings into clinical practice, particularly in optimizing treatment timing.

Further investigations involving extensive multicenter cohorts are needed to confirm the clinical relevance of HMGB1 and improve therapeutic approaches. Investigating its interplay with other biomarkers and pathways may yield insights into more comprehensive diagnostic and therapeutic approaches. Ultimately, integrating HMGB1 into clinical frameworks holds the potential to transform the management of AKI, improving patient outcomes and alleviating the disease burden.
